# Prevalence of “HIV/AIDS related” parental death and its association with sexual behavior of secondary school youth in Addis Ababa, Ethiopia: a cross sectional study

**DOI:** 10.1186/1471-2458-14-1120

**Published:** 2014-10-30

**Authors:** Takele Menna, Ahmed Ali, Alemayehu Worku

**Affiliations:** School of Public Health, College of Health Sciences Addis Ababa University, Addis Ababa, P.O. Box 33412, Ethiopia

**Keywords:** Secondary school, Youth, Orphan, Non-orphan, Multiple sexual partners, HIV/AIDS

## Abstract

**Background:**

Human immunodeficiency virus infection is a global crisis that represents a serious health threat, particularly among younger people. Various studies show that both orphan and non-orphan adolescents and youths experience vulnerability to HIV. Nevertheless, the findings hitherto are mixed and inconclusive. The aim of this study, therefore, was to assess the prevalence of parental death and its association with multiple sexual partners among secondary school students for evidence based interventions.

**Methods:**

A cross-sectional study was conducted among secondary school youth in Addis Ababa, Ethiopia. A multistage sampling technique was used to select a representative sample of 2,169 school youths. Sexual health behavior related data were collected using self-administered questionnaire. Binary logistic regression was employed to examine the relation between parental death and multiple sexual partners.

**Results:**

Among the 2,169 eligible study participants 1948 (90%) completed the self-administered questionnaires. Of those 1,182(60.7%) were females. The overall prevalence of parental death was 347(17.8%.) with 95% CI (16.2%, 19.6%). The HIV/AIDS proportionate mortality ratio was 28% (97/347).

A multivariate logistic regression analysis showed that high HIV/AIDS related knowledge (AOR = 0.39; 95% CI, 0.18-0.84), positive attitude towards HIV prevention methods (AOR = 0.48; 95% CI, 0.23-0.97), being tested for HIV (AOR = 0.52; 95% CI, 0.31-0.87) and chewing Khat (AOR = 2.59; 95% CI,1.28-5.26)] were significantly associated with having multiple sexual partners among secondary school youths.

**Conclusions:**

Significant proportion of secondary school youths had lost at least one parent due to various causes. High knowledge of HIV/AIDS, positive attitude towards ‘ABC’ rules for HIV prevention, being tested for HIV and chewing khat are more likely to be factors associated with multiple sexual partnership among secondary school students in Addis Ababa.

Therefore, the school based interventions against the HIV/AIDS epidemic should be strengthened with particular emphasis on the effects of HIV/AIDS related knowledge, attitude towards preventive measures, mechanisms for improving HIV Counseling and Testing coverage and the associated prevailing risk factors.

## Background

Human immunodeficiency virus (HIV) infection is a global crisis that represents a serious health threat, particularly among younger people [1]. Ethiopia is among the countries most affected by the HIV epidemic. With an estimated adult prevalence of 1.5%, it has a large number of people living with HIV (approximately 800,000) and about 1 million AIDS orphans [2]. In countries with high HIV prevalence and the resultant high adult mortality rate there will be higher incidence and prevalence of orphans [3].

While AIDS-related deaths are fortunately declining due to biomedical interventions supported through social protection mechanisms, a study on the Global Burden of Diseases, Injuries, and Risk Factors showed that in 2010 AIDS remained to be the fifth-leading cause of disease-related deaths, with an estimated 1.5 million AIDS-related deaths [3].

HIV/AIDS directly impacts on the lives of approximately 20 million children worldwide and an estimated 16.6 million children have lost one or both parents due to the disease [4, 5].

The negative effects of the AIDS epidemic are felt most severely in some of the world’s poorest countries in sub-Saharan Africa. One of its effects has been an increase in the number of orphaned children [6–8].

It is estimated that for each woman who dies of AIDS in Africa, two children will be orphaned [9]. The epidemic in Africa puts children at risk physically, emotionally and economically. These challenges may further predispose these children at heightened risk of prolonged mental and behavioral problems [10].

A study demonstrated that AIDS-orphans have more depression, peer problems, and behavior problems than other groups, but no difference in anxiety [11]. Other study conducted in Kenya reported, although orphans are at higher risk for psychosocial problems that may affect their self-efficacy for safer sex practices more than non-orphans, no difference in HIV risk indictors were identified [12].

Nevertheless, various studies conducted in Sub-Saharan African countries have shown that orphaned children experience particular vulnerability to contract HIV [13, 14].

A Meta-analysis of six studies (n =19,140) comparing HIV-positive sero status in orphaned versus non-orphaned youths indicated that there is significantly greater HIV prevalence in orphaned participants compared with non-orphans [7].

The results of other four studies showed that orphans were significantly more likely to have experienced sexual debut than non-orphans (Cote d’Ivoire, Lesotho, Mozambique and Tanzania) [15].

The importance of concurrent sexual partnerships in the transmission of HIV has been highlighted in a study. A number of social drivers of concurrency, including factors which may be relevant to orphan hood, such as the need for partners in order to reduce fears of rejection or of being alone and concurrent partners as an economic strategy or necessity were reported from a qualitative data [16].

Various studies have identified multiple sexual partnerships by both men and women, particularly overlapping or concurrent partnerships, to lie at the root of the HIV/AIDS epidemic in sub-Saharan Africa [17].

A study from South Africa showed that parental death among female participants was significantly associated with HIV-positive status, ever having had oral sex, ever having had vaginal sex and having more than 1 sex partner during the past year [18].

Another study demonstrated that female adolescent orphans in urban Zimbabwe were at higher risk of HIV and HSV-2 infection than non-orphans because of their higher likelihood of having had multiple sexual partners, having used condoms more inconsistently, and having experienced forced sex [13].

As the National Surveys of four African countries revealed, in overall, 12 percent of boys and about 5 percent of girls reported that they had two or more sexual partners during the year before the survey. On the other hand, a higher proportion of older adolescents who were sexually active in the 12 months before the survey reported to have had two or more partners in Malawi [19].

In addition, as evidences suggest a substantial proportion of young people continue to engage in risky sexual behaviors. For instance, according to the Health Impact Evaluation done in Ethiopia in 2008, 48%, 50% and 58% of women aged 15-24 reported consistent condom use, limiting sexual intercourse with one uninfected partner and abstaining from sex , respectively [20].

Many young people rule out the option of abstinence as an AIDS prevention methods . But a study showed that for many giving up sexual intercourse to prevent AIDS would be “impossible” [20]. Although young people believe that condoms effectively prevent STDs, only small proportions of college students have adopted measures such as condom use [21].

A systematic review of school based interventions to prevent STI/HIV in sub-Saharan Africa showed that behavior change was least likely to occur and changes in favor of abstinence and condom use were very much influenced by pre-intervention sexual history [22].

Evidences from various studies showed that there are some associations between using substances like alcohol, tobacco and Khat and risky sexual behaviors [13, 23].

As studies from sub-Saharan Africa show, there is a link between alcohol drinking and a risk for concurrent sexual partnership [24, 25]. In Kenya, orphaned children, especially maternal orphans, reported more alcohol use and risky sex [26].

Khat is a strong stimulant that causes mild to moderate psychological dependence, although not as strong as that of alcohol and tobacco. Some of the possible effects of chewing Khat comprise increased levels of energy, increased self-esteem, euphoria, increased libido, excitement and tendency to social interaction [27, 28].

Khat use was positively associated with being male, alcohol use, not having comprehensive knowledge on HIV and viewing sexual films. Khat is widely consumed among the youths of Ethiopia as shown by several prevalence studies [27, 29].

Young orphans may be at elevated risk of HIV including involvement in sexual activities with multiple partners, because of being more likely to lack adult guidance than their peers. The evidence on whether this is the case lacks clarity or shows mixed results [15]. Thus, there are still critical gaps to be bridged regarding the required efforts for better support of children affected by HIV/AIDS.

Furthermore, young people in various countries continue to engage in high risk sexual behaviors despite the presence of robust HIV prevention strategies aimed at reducing risky sexual behaviors [30]. High-risk behaviors, such as having multiple sexual partners, alcohol and substance use, unplanned pregnancies and unprotected sexual activities of children and adolescents are major concerns for many countries of the world [30–34].

Therefore, the objective of this study was to contribute to interventions serving orphans by exploring the prevalence of parental death and its association with multiple sexual partners of school youth aged 15 and above years in a major urban setting in Ethiopia.

## Methods

### Study setting

A secondary school based cross-sectional study was conducted from February to March, 2013 in Addis Ababa, the capital of Ethiopia. According to the national census report of 2007, the projected population of Addis Ababa for the year 2012 was 3,048,631 and among those 52 .4% were females [35, 36]. The City is administratively divided into ten sub-cities (Kifle- Ketemas) and one –hundred and sixteen districts/Woredas [36]. There were 163 secondary schools (both government and non-government schools) in Addis Ababa City Administration. The number of students enrolled in the year 2012/2013 in the secondary schools was 136,636(53.9% females) [37]. In addition, there were 14, 893 (44.4% female) teachers in primary schools and 5,651 (17.2% female) teachers in secondary schools of the City for the same year [37].

### Sample size and sampling procedure

As this study is part of a large scale study with different objectives that comprise assessing the trend of HIV/AIDS related mortality among teachers, examining the proportion of students who lost one or both parents due to HIV/AIDS related causes, determinants of risky sexual practice and HCT uptake among students and teachers and exploring the effects of peer education intervention on sexual behavior of students. The sample size of this study was determined using the formula for single population proportion estimation by taking 33% as a proportion of school youth involved in risky sexual behavior based on findings of previous studies [38–40], and 3% of absolute precision with 95% confidence interval. Non-response rate in this study was estimated to be 15% and a design effect of 2 was assumed. Hence, an overall sample size of 2,169 students from randomly selected 15 public secondary schools in Addis Ababa participated in the study [37].

The adequacy of the sample to run internal comparison for assessing the association between parental death and multiple sexual partner among students was checked by calculating sample size using double proportion formula which gave a sample size less than the one mentioned above. The study population was randomly selected using multi-stage cluster sampling method. There are 10 sub-cities in Addis Ababa City Administration, Ethiopia. The number of participant public secondary schools from each sub- city was decided proportionately.

All the schools in each sub-city that satisfy the inclusion criteria were listed alphabetically and separately. Systematic sampling technique was used to select participating schools. Lists of all students in sections of grade 9-12 in each selected school were collected before the survey. The total sample size was distributed to each school proportionately to the size of their student population.

The participant students were those from randomly selected sections who were 15 and above years old and provided consent.

### Data collection and quality control

Data were collected using self administered questionnaire that had several sections dealing with various socio demographic and sexual behaviors related variables. The questionnaire was primarily prepared in English and then translated in to the local official language, Amharic for better understanding in its actual administration. The questionnaire was pre tested in two secondary schools that were not selected for the study. Then, the necessary correction was made in language and content for better clarity and more understandability. Data collection was conducted by 12 diploma graduate nurses under the supervision of two senior health professionals who were also working as instructors in different health colleges.

Two days training was given for the data collectors and supervisors by the principal investigator. The data quality was assured by giving training to data collectors and supervisors as well as conducting close, immediate and daily supervisions. The collected data were checked for completeness and consistency by supervisors and the principal investigator every day.

The final modified questionnaire was used to generate key information from the study participants.

Accordingly, the participant students were responded to the following sexual behavior related variables:whether they faced parental death due to any cause, parental death related to HIV/AIDS or not, sex, age, HIV/AIDS related knowledge assessing questions, attitude towards “ABC” rules as HIV prevention methods assessing questions, history of sexual exposure and HCT uptake, history of condom use and multiple sexual partners in previous year, whether they had habits of using substances like drinking alcohol, chewing khat and smoking cigarettes or not.

Furthermore, the choice of variables included in bivariate analysis was based on theoretical knowledge and statistical significance (p-value < 0.05).

Ethical clearance was obtained prior to the study from the Institutional Review Board of the College of Health Sciences of the Addis Ababa University. Official letter of cooperation was written from the School of Public Health of Addis Ababa University to Addis Ababa City Administration Education Bureau. The Education Bureau wrote letters of cooperation to all concerned including the selected study schools from all the ten sub cities.

Finally, verbal consent of individual participants was obtained after being fully informed the purpose of the study and procedures of data collection. No name or other identifying information were included in the data collection to ensure confidentiality and anonymity.

### Data analysis

Data were entered and cleaned using Epi-Info software version 3.5.4 and then transported to SPSS software version 20 for analysis. Age, sex, orphan-hood due to any cause and due to HIV/AIDS related causes, HIV/AIDS related knowledge, attitude towards “ABC” rules to prevent HIV, prevalence of HCT uptake, substance use and other sexual behavior related variables were chosen for statistical analyses. Descriptive statistics such as frequencies and proportions were used to describe the study population in relation to relevant variables. Bivariate analysis between orphan-hood as a main exposure and history of having multiple sexual partners/one of UNAIDS indicators of risky sexual behaviors/as dependent variable was employed.

Then, a stepwise multivariate logistic regression model was used to assess the associations between the outcome and orphan hood as well as other predictors for multiple sexual partners. The variables in multivariate analysis were chosen based on existing theoretical knowledge on the variables and statistical significance found during bivariate analyses.

## Results

Among 2169 eligible study subjects 1948 school youth (90%) completed the self administered questionnaires. Of those study participants 1,182(60.7%) were female. The vast majority (80.5%) of the respondents were in the age group of 15-18 years, and only 19.0% and 0.5% were 19-24 and above years respectively. Ninety- five percent of the students were single. However, 484 (25%) responded that they had sexual partners during February and March, 2013 and among them 417 (21.5%) ever practiced sexual intercourse. Of the students who had history of sexual intercourse in preceding year of the survey 154 (54.4%) and 175(62.3%) had two or more sexual partners and history of inconsistent condom use, respectively. The age and sex distributions of non-respondents 221 (10.2%) were believed to be similar to that of the respondents because of being in similar schools and grade levels. Besides, this fact was observed from the annual report prepared by the Federal Ministry of Education of Ethiopia for the year 2012/13 and data collected from each participant school through desk review during preliminary assessments [37].

### Prevalence of parental death

The prevalence of any parental death among the study subjects was 347 (17.8%) with 95% CI (16.2%, 19.6%), (Table 1). Among those 211(60.8%), 70 (20.2%) and 66 (19.0%) reported that their fathers, mothers and both parents respectively died due to various causes. Moreover, the HIV/AIDS proportionate mortality ratio was 28% (97/347).

**Table 1:** **Distribution of socio-demographic back ground by parental death among school youth in Addis Ababa Ethiopia (N = 1948), 2012/13**

Variables	Both parents are alive n = 1601 (%)	At least one of the parents deceased n = 347 (%)	Father deceased n = 211 (%)	Mother deceased n = 70 (%)	Both parents deceased n = 66 (%)
**Sex**					
Male	635(39.7)	131(37.8)	80(37.9)	21(30.0)	30(45.5)
Female	966(60.3)	216(62.2)	131(62.1)	49(70.0)	36(54.5)
**Age category**					
15-18	1301(81.5)	263(75.8)	160(75.8)	56(80.0)	47(71.2)
>18 years	295(18.5)	84(24.2)	51(24.2)	14(20.0)	19(28.8)
**Education**					
Over all	1599(82.2)	346(17.8)	211(10.9)	69(3.6)	66(3.4)
Grade 9-10	641(40.1)	140(40.6)	87(41.2)	25(36.2)	30(45.5)
Grade 11-12	958(59.9)	205(59.4)	124(58.8)	44(63.8)	36(54.5)
**Percentage of over all parental death**		347 (17.8)	211 (60.8)	70 (20.2)	66 (19.0)
**Proportion of HIV/AIDS related orphans**		97(4.99)	51(52.6)	19 (19.6)	27(27.8)
**Ever had sex**					
No	1271(79.8)	249(72.4)	154(74.0)	48(68.6)	47(71.2)
Yes	322(20.2)	95(27.6)	54(26.0)	22(31.4)	19(28.8)
**Had sex in previous year**					
No	90(29.8)	19(20.9)	11(21.2)	7(36.8)	1(5.3)
Yes	212(70.2)	72(79.1)	41(78.8)	12(63.2)	18(94.7)
**No. of sexual partners in previous year**					
One	97(46.0)	32(44.4)	19(46.3)	3(25)	9(50.0)
Two or More	114(54.0)	40(55.6)	22(53.7)	9(75.0)	9(50.0)
**Condom used**					
Consistently	83(39.7)	23(31.9)	11(26.8)	3(25.0)	8(44.4)
Inconsistently	126(60.3)	49(68.1)	30(73.2)	9(75.0)	10(55.6)

In order to assess the associations between having multiple sexual partners and various associated factors like orphan hood, bivariate logistic regression analysis was done (Table 2). Among the variables selected as multiple sexual partner predictors, having high knowledgeable on HIV/AIDS related topics (OR = 0.45; 95% CI, 0.22-0.93), showing positive attitude towards “ABC” rules for HIV prevention (OR = 0.48; CI, 0.25-0.95), being ever tested for HIV (OR = 0.53; 95% CI, 0.33-0.85), and chewing Khat (OR = 1.80; 95% CI, 1.0-3.26), showed positive associations. However, being female (OR = 1.22; 95% CI, 0.76-1.96) age greater than 18 (OR = 0.79; 95% CI, 0.48-1.31), orphan due to any cause (OR = 1.06; 95% CI, 0.62-1.82) and HIV/AIDS related orphan hood (OR = 0.90: 95% CI, 0.40-2.05) did not show statistically significant associations (Table 2).

**Table 2:** **Sexual behavior and associated factors among Secondary school students in Addis Ababa, Ethiopia (N = 417), 2012/13**

Variable	No of sexual partners in previous year			
	Two or more n(%)	Only one n(%)	Un adjusted OR (95% CI)	AOR (95% CI)
**Sex**				
Male	75(47.8)	82(52.2)	1.0	1.0
Female	54(42.9)	72(57.1)	1.22(0.76-1.96)	1.44(0.86-2.40)
**Age in completed years**				
15-18	84(44.2)	106(55.8)	1.0	1.0
>18	45(50.0)	45(50.0)	0.79(0.48-1.31)	0.76(0.44-1.32)
**Lost at least one parent due to any cause**				
No	97(46.0)	114(54.0)	1.0	1.0
Yes	32(44.4)	40(55.6)	1.06(0.62-1.82)	1.18(0.59-2.36)
**Lost at least one parent due to HIV/AIDS related cause**				
**No**	117(45.3)	141(54.7)	1.0	1.0
**Yes**	12(48.0)	13(52.0)	0.90(0.40-2.05)	0.81(0.28-2.35)
**Level of HIV/AIDS related knowledge**				
Low	22(62.9)	13(37.1)	1.0	1.0
High	107(43.1)	141(56.9)	0.45(0.22-0.93)	0.39(0.18-0.84)
**Attitude towards ‘ABC’ rules of HIV prevention**				
Negative	25(61.0)	16(39.0)	1.0	1.0
Positive	104(43.0)	138(57.0)	0.48(0.25-0.95)	0.48 (0.23-0.97)
**HCT up-take**				
No	77(53.5)	67(46.5)	1.0	1.0
Yes	52(37.7)	86(62.3)	0.53(0.33-0.85)	0.52(0.31-0.87)
**Drink alcohol**				
No	75(55.1)	61(44.9)	1.0	1.0
Yes	79(53.7)	68(46.3)	0.95(0.59-1.51)	0.66(0.38-1.15)
**Chew khat**				
No	113(51.4)	107(48.6)	1.0	1.0
Yes	40(65.6)	21(34.4)	1.80(1.0-3.26)	2.59(1.28-5.26)

Furthermore, a multivariate logistic regression was done to assess the independent associated factors of having multiple sexual partners in previous year of the survey among secondary school students. High HIV/AIDS related knowledge (AOR = 0.39; CI, 0.18-0.84), positive attitude towards HIV prevention methods (AOR = 0.48; 95% CI, 0.23-0.97), ever being tested for HIV (AOR = 0.52; 95% CI 0.31-0.87) and chewing Khat (AOR = 2.59; 95% CI,1.28-5.26) were significantly associated with having multiple sexual partners among secondary school youths during multivariate logistic analysis ( Table 2).

## Discussion

This study was conducted among secondary school students in Addis Ababa. The high proportions of age category from 15-18 and female population in the sample were in agreement with the characteristics of current student population in secondary schools in Ethiopia [37].

The findings of this study showed that the prevalence of sexual initiation among secondary school youths is 21.5%. This finding is almost similar to that of the previous studies conducted among high school adolescents in the country [30, 33]. An individual’s level of risk for contracting HIV was assessed through one of the UNAIDS proposed core indicators or having multiple sexual partners in previous 12 months.

The proportions of having multiple sexual partners among the study participants in general and those who ever had sexual intercourse in preceding 12 months of the survey were 7.9% and 54.4%, respectively. In addition, one of the important findings of this study is the prevalence of inconsistent condom use among sexually active school youth in preceding year is 62.3%. These findings are in harmony with the findings of studies conducted previously in Ethiopia and other African countries [13, 19, 20].

As the results of this study reveal, nearly one- fifth (17.8%) of the study subjects experienced death of at least one parent. Most of the orphaned students had lost their fathers (10.9%). This finding is also in line with the findings of the research that had analyzed the Demographic and health surveys and related household surveys of ten African countries (Benin, Chad, Congo (Brazza-ville), Côte d’Ivoire, Lesotho, Malawi, Mozambique, Tanzania, Uganda and Zimbabwe) [41].

Although the magnitude of the prevalence of orphan-hood is very significant, our finding is slightly lower than those reported previously from Zimbabwe in 2008 [13]. However, among the students who lost one or both parents due to various causes, 28% reported that they lost their parents due to HIV/AIDS.

The study also showed that the proportion of students who had multiple sexual partners in previous year didn’t have statistically significant variation in age and sex. Nonetheless, these findings are not consistent with the findings from National surveys of four African countries that showed differences in sex and age groups regarding exposure to multiple sexual partners, and other studies done in South Africa and Burkina Faso that indicated variations in sexual behavior among different sex and age groups [18, 19, 42].

The hypothesis on risky sexual behavior that states, students who lost at least one parent due to any cause, including HIV/AIDS are more exposed to HIV because of the likelihood that the orphaned youths could face more economic challenges and/or lack parental guidance was not supported by our findings. This is also in agreement with the study done in a neighboring country, Kenya [12]. But our findings are not in line with findings of the previous studies conducted in some other countries [5, 13, 15]. This could be explained in terms of the difference in economic statuses of surveyed countries and effectiveness of intervention programs to care and support orphaned children and adolescents.

The most significant findings of this study are having high knowledge on HIV/AIDS, positive attitude towards the common preventive methods “ABC” rules and being tested for HIV had shown an intended positive associations with having multiple sexual partners among school youths during both bivariate and multivariate analyses. However, these observations are not in accordance with findings from different countries that reported “ both in school and out of school youth are experiencing risky sexual behavior and do little to prevent HIV and other STIs despite their high knowledge on HIV/AIDS and positive attitude towards its prevention methods” [20, 22]. On the other hand, in many instances, low knowledge of HIV transmission methods and negative attitude to preventive measures like using condoms are associated with sexual behavior among adolescents who are most at risk [42].

Furthermore, having multiple sexual partners among school youth was not associated with parental loss due to HIV/AIDS or any other cause during both bivariate and multivariate analyses. But being tested for HIV and Chewing Khat were significantly associated with multiple sexual partnerships among secondary school youths.

As the primary aim of HCT is preventive, the finding of this study is in agreement with the findings of studies conducted in many other African countries that revealed the significant effects of HCT on reducing the magnitude of risky sexual practice [43–45]. On the other hand, our findings are not in line with the findings of a study that revealed individuals who tested negative were more likely to report multiple sexual partnership and intercourse with non-primary sexual partners [46].

The observed association between having multiple sexual partners and chewing Khat could be because of the effects of stimulant substance found in Khat that may impair the ability of the mind for right judgment by temporally decreasing the level of fear against any risk factor including HIV. In addition, comparing with their peers those youths who are actively involved in chewing Khat are more likely to drink alcohol and smoke cigarettes which could increase the probability of indulging into risky sex. Similar associations were also observed in the findings of various studies conducted in different countries [13, 23, 27–29, 43].

Nevertheless, as the information for this study were obtained from self reports of the study participants and there were no opportunities to cross check the opinion from other sources, the observed associations could be assessed further.

Furthermore, as the study design was cross sectional, although it is strong to establish associations, the probability of identifying the true effect of orphan hood on having multiple sexual partners is weak. In addition, since the study participants were only from secondary schools, there might be a challenge to implement the expected interventions based on the findings of this study to the primary school and college students in various settings.

However, the random selection of study participants, large sample size, and data collectors and supervisors being health professionals with diploma and above educational background can be taken as the significant strengths of this study.

## Conclusions

In general, about one- fifth of school youths in Addis Ababa experienced parental death. High knowledge of HIV/AIDS, positive attitude towards “ABC” rules for HIV prevention, being tested for HIV and chewing khat are more likely to be factors associated with multiple sexual partnerships of both orphaned and non-orphaned secondary school students in Addis Ababa.

The education sector and its stake- holders should strengthen the current intervention strategies against the HIV/AIDS epidemic by giving due consideration to the intended outcomes of the above associated factors of sexual behaviors among school youths through mini media, HIV/AIDS clubs, peer education, school community conversation and the like.

Finally, nationally representative research on the same problem and its outcome for better and multi dimensional interventions against the fatal epidemic of HIV/AIDS can also have paramount importance.
